# Precision of Measurements Performed by a Cadre of Anthropometrists Trained for a Large Household Nutrition Survey in Ethiopia

**DOI:** 10.1093/cdn/nzaa139

**Published:** 2020-08-21

**Authors:** Cami Moss, Desalegn Kuche, Tesfaye Hailu Bekele, Mihretab Salasibew, Girmay Ayana, Andinet Abera, Solomon Eshetu, Alan D Dangour, Elizabeth Allen

**Affiliations:** London School of Hygiene and Tropical Medicine, London, United Kingdom; Ethiopian Public Health Institute, Addis Ababa, Ethiopia; Ethiopian Public Health Institute, Addis Ababa, Ethiopia; London School of Hygiene and Tropical Medicine, London, United Kingdom; Ethiopian Public Health Institute, Addis Ababa, Ethiopia; Ethiopian Public Health Institute, Addis Ababa, Ethiopia; Ethiopian Public Health Institute, Addis Ababa, Ethiopia; London School of Hygiene and Tropical Medicine, London, United Kingdom; London School of Hygiene and Tropical Medicine, London, United Kingdom

**Keywords:** nutrition, anthropometric data, anthropometry, precision, standardization

## Abstract

**Background:**

Well-trained anthropometrists are essential for the delivery of high-quality anthropometric data used to evaluate public health nutrition interventions. Scant data are currently available on the precision of data collected by large teams of anthropometrists employed for nutrition surveys in low-income country settings.

**Objectives:**

The purpose of this study was to assess the precision of child midupper arm circumference (MUAC) and length/height measurements taken by fieldworkers training for nutrition survey deployment.

**Methods:**

Following 3 d of training, an anthropometry standardization exercise was conducted in small teams of trainees at 7 sites in the Amhara region of Ethiopia. In groups of 2–4, trainee anthropometrists (*n* = 79) each measured 16 children aged 6–47 mo (*n* = 336) twice for MUAC and length/height. Both intraobserver and interobserver precision were analyzed using technical error of measurement (TEM), relative TEM, coefficient of reliability (*R*), and repeatability metrics. Bland–Altman limits of agreement were calculated for intraobserver measurements.

**Results:**

Intraobserver TEM was between 0.00 and 0.57 cm for MUAC (Bland–Altman 95% limits of agreement: −0.50 to 0.54 cm) and between 0.04 and 2.58 cm for length/height measurements (Bland–Altman 95% limits of agreement: –1.43 to 1.41 cm). Interobserver TEM was between 0.09 and 0.43 cm for MUAC and between 0.06 and 2.98 cm for length/height measurements. A high proportion of trainees achieved intraobserver *R* >0.95 (MUAC: 95%; length/height: 97%). Most teams also achieved interobserver *R* >0.95 (MUAC: 90%; length/height: 95%).

**Conclusions:**

Large numbers of anthropometrists (>75) in low-income settings can attain satisfactory precision in anthropometry following training and standardization. These protocols permit researchers to assess trainees, identify individuals who have not achieved the desired level of precision, and retrain or adjust roles prior to survey deployment.

## Teaser

Well-trained anthropometrists are essential for the delivery of high-quality anthropometric data used to evaluate public health nutrition interventions. Large numbers of anthropometrists in low-income settings can attain satisfactory precision in length/height and arm circumference measurements through training and standardization.

## Introduction

High-quality measurement of child anthropometry is required to evaluate the effectiveness of nutrition interventions at scale. Measurement precision is an essential indicator of data quality, and large variation between observers may increase measurement bias (1). Yet large-scale household nutrition surveys rely on trainee anthropometrists who may have limited or no prior experience in measuring children.

Standardization of anthropometric techniques is recognized as one way to achieve high precision among a large number of observers (2). Apart from major growth studies such as NHANES (3) or WHO's Multicentre Growth Reference Study (4), only a few studies have detailed the approach and methods used to standardize anthropometric measurement techniques for infants and young children (5–13). Such methods are of special relevance to program evaluation studies, for which growth is one of multiple data indicators collected and for which anthropometrists (who also double as household interviewers) must also receive training in selection methods, questionnaires, and interviewing techniques (14). Other constraints may include the very large number of child participants, measurements, and multiple activity sites required to standardize large numbers of trainees in anthropometry; these conditions may preclude the availability of a sufficient number of expert, or “gold standard,” anthropometrists to estimate trainee accuracy (2). This study set out to develop and document solutions to these challenges to improve the quality of the survey data and help fill the evidence gaps.

The Sustainable Undernutrition Reduction in Ethiopia (SURE) program is a government-led integrated health and agriculture intervention that aims to improve child complementary feeding and dietary diversity. The main program components comprise interpersonal counseling on child feeding and dietary diversity by community-based health and agriculture extension workers, men's and women's group dialogues, mass media messages, and support for local multisectoral nutrition coordination committees. SURE is implemented by the Federal Ministries of Health and Agriculture and evaluated by the Ethiopian Public Health Institute (EPHI) in partnership with the London School of Hygiene and Tropical Medicine.

This article describes the methods used to conduct the anthropometry standardization exercise under “real-world” survey conditions and assesses intraobserver and interobserver precision in a large group of anthropometrists employed to collect data as part of the SURE program evaluation baseline study.

## Methods

### Training

The baseline survey training for anthropometrists was based primarily at EPHI in Addis Ababa, Ethiopia, and took place over 3 wk in April 2016. Within the training period, anthropometric training and standardization were conducted over a total of 5 d.

We trained a total of 79 trainees and team supervisors in anthropometry for 3 d prior to beginning the 2-d standardization exercise. The objectives of the training were to introduce the history and purpose of anthropometry, to teach the techniques and operational procedures for each type of anthropometric measurement, and to have trainees practice measurements with the oversight and correction of specialists. On the first day, we used WHO materials to teach anthropometry using a demonstration video, detailed descriptions of operational procedures and measurement techniques, and group discussion of common sources of error (15). On the second day, trainees were split into 3 groups, and researchers demonstrated correct anthropometric techniques during the morning session. Trainees spent the afternoon practicing measurements for the first time by splitting into teams of 4 to take height, weight, and MUAC measurements on one another. The researchers monitored each group and provided correction throughout.

On the third day of training, teams of trainees left the central training facility and traveled to 1 of 7 *kebeles* (subdistricts) to practice weight, length/height, and MUAC measurements of children aged 6–47 mo. Within small teams of 3 or 4, each trainee repeated measurements twice on each of 8 different children during the course of the training day. One researcher at each kebele monitored and provided correction to a maximum of 12 trainees.

All baseline survey anthropometrists and supervisors had at minimum a bachelor's degree in a public health or nutrition-related field and passed a written examination.

### Data collection teams and procedures

After 3 d of anthropometry training and practice, a dedicated 2-d standardization exercise was undertaken. During the exercise, anthropometric data were collected at 7 different sites within Basona Worena *woreda* (district) in Amhara region, Ethiopia. Each anthropometrist—in a team of 2–4 trainees—took 2 MUAC measurements and 2 length/height measurements for each of the same 16 children. Weight measurements were not prioritized for inclusion in the standardization exercise due to low expected variation (10).

*Imprecision* is defined as the variability of repeated measurements and is due to both intra- and interobserver differences in measurement (16). *Unreliability* is a combination of imprecision and undependability; the latter is defined as variability that is due to physiological variation and may be captured by taking a child's measurements repeatedly at different time points (1). In this study, constraints on volunteer caregivers meant that it was impracticable to organize the first measurement of a child 1 d and the second measurement of the same child on the second day. Therefore, this study assessed precision and not reliability.

One member of the research team supervised 3 teams assigned to each of 7 different kebele health posts, or sites. Twenty-one anthropometry teams comprised between 2 and 4 individuals who worked in pairs or trios, switching roles as primary measurer, assistant, or recorder (as required).

A local health extension worker was responsible for recruiting caregivers of children aged 6–47 mo at each of the health posts (*n* = 336). Children were recruited to attend 1 morning or 1 afternoon session. Four children were assigned to each anthropometry team in the morning, and 4 new children were assigned to the same team in the afternoon. This procedure was repeated on the second day with new sets of children. Children who were highly anxious and uncooperative were released during the first attempted measurement and replaced with another child.

In each group of 4 children per half-day session, each child's MUAC measurement was taken once by each member of the anthropometry team acting as primary measurer with 1 other team member assisting to position the child. The process was then repeated for each child's length/height measurement. A third team member (or volunteer, in the case of 2 team members only) recorded length/height measurements. Trainees were instructed to record results independently and not to share them with other team members, except the designated recorder for length/height measurements.

Upon completion of the first set of 1 MUAC and 1 length/height measurement for each of the 4 children per session, anthropometrists submitted individual data recording sheets to a researcher on site prior to beginning the second set of measurements. This was intended to help prevent recall. Then the second set of measurements were taken. This procedure was repeated for each of the 4 sessions during the 2-d standardization exercise, resulting in each anthropometry team measuring a total of 16 children per team and per individual trainee.

### Measurements

We used color-coded MUAC strips produced for use in Ethiopian nutrition surveys and provided to EPHI by UNICEF Ethiopia (Addis Ababa, Ethiopia). A child's left arm was measured by flexing the elbow to a right angle and marking the midpoint between the acromium process and the tip of the elbow. The arm was then released and the MUAC strip was fitted around the midpoint of the arm and the measurement was recorded to the nearest 0.1 cm.

To measure length/height, we used a portable measuring board (UNICEF Supply Division, Copenhagen, 2016). Children aged <24 mo or <86 cm had length measured horizontally, whereas those aged >24 mo or >86 cm had height measured standing upright. Where possible, the board was placed on a flat surface, and for height measurements, the backboard was supported by a wall. All children were measured with their heels together and touching the board, knees straight, and their heads positioned in the Frankfort horizontal plane. Standing children were also positioned with their buttocks against the board and their spines straightened. Measurements were recorded to the nearest 0.1 cm.

### Statistical analysis

We analyzed precision using several related metrics. Technical error of measurement (TEM) is the most commonly used for anthropometric measurements. We calculated the TEM as the square root of the measurement error variance. This is a measure of within-subject variability when measures are taken repeatedly (1). For 2 observers, TEM is calculated as follows:

(1) $$\begin{eqnarray*}
{\rm{TEM}} = \sqrt {\frac{{(\Sigma {{\rm{\mathit{ D}}}^2})}}{{2{\rm{\mathit{ N}}}}}}
\end{eqnarray*}$$

where *D* is the difference between measurements, and *N* is the number of individuals measured.

For more than 2 observers, TEM was calculated as follows (1):

(2) $$\begin{eqnarray*}
{\rm{TEM}} = \sqrt {((\Sigma _1^N((\Sigma _1^K{M^2}) - ({{(\Sigma _1^KM)}^2}/K)))/(N(K - 1))}
\end{eqnarray*}$$

where *K* is the number of observers, *N* is the number of individuals measured, and *M* is the measurement.

We also calculated the percentage TEM, or relative TEM, to enable comparison across different units and studies:

(3) $$\begin{eqnarray*}
{\rm{Relative}}\,{\rm{TEM}} = \left( {\frac{{TEM}}{{Mean}}} \right) \times 100
\end{eqnarray*}$$

For intraobserver precision, we calculated the coefficient of reliability (*R*), which estimates the proportion of intersubject variance that is not due to measurement error, as follows:

(4) $$\begin{eqnarray*}
{\rm{\mathit{ R}}} = 1 - \left( {\frac{{{{(TEM(Intra))}^2}}}{{{{(S)}^2}}}} \right)
\end{eqnarray*}$$

where *S* is the within-subject SD among the children measured.

For interobserver precision, we calculated *R*:

(5) $$\begin{eqnarray*}
{\rm{\mathit{ R}}} = 1 - \left( {\frac{{{{(TEM(Inter))}^2}}}{{{{(S)}^2}}}} \right)
\end{eqnarray*}$$

where *S* is the within-subject SD among the children measured.

We calculated repeatability as the value representing the maximum distance between 2 separate measurements for 95% of subjects. This is calculated as 1.96$\sqrt {2s} $, where *s* is within-subject SD (TEM), or 2.77 multiplied by TEM (17).

Bland–Altman analyses assess the magnitude of the disagreement (error and bias) between the first and second measurements recorded by the same anthropometrist on the same child. Using the Stata package *agree* (StataCorp) (18), Bland–Altman plots were generated to graphically depict these differences and to calculate the 95% limits of agreements as a reference interval between which lie all but 5% of the observed differences in intraobserver measurement (17, 19).

All calculations were completed in Stata version 14.0 (20). All data are available in **Supplemental Table 1**.

### Additional training

After statistical analysis was completed at the end of the 2-d standardization exercise, results were presented to trainee anthropometrists. Individuals and members of measurement teams who scored *R* coefficients <0.95 in any set of measurements were given additional instruction by the training staff (2).

### Ethics

The SURE baseline study was approved by the Scientific and Ethical Review Committee at EPHI (Ref: SERO-54-3-2016) and the ethics committee of the London School of Hygiene and Tropical Medicine (Ref: 10,937). The study was conducted in accordance with preapproved protocols. By approval of both institutional review boards, informed oral consent was given by caregivers of young children and witnessed by the local health extension worker as a result of the known reticence of illiterate persons in Ethiopia to sign or thumbprint unfamiliar documents.

## Results

A total of 79 observers (81% male; mean age: 28.8 ± 4.7 y), including trainee anthropometrists and team supervisors, completed the standardization exercise. There were 21 anthropometry teams, of which there were 17 teams of 4 observers, 3 teams of 3 observers, and 1 team of 2 observers. Each of the observers acted as the primary measurer 4 times per child and 64 times in total during the 2-d exercise.

Each team measured 16 children, for a total of 336 children aged 6–47 mo. Children were measured by each member of the anthropometry team to which they were assigned, for a minimum of 8 and a maximum of 16 separate measurements.

Details of the participants are shown in **Table 1**.

**TABLE 1:** Number of observers and participant characteristics by standardization site^1^

		Participants (*n*)				
Site number (kebele)	Observers (*n*)	Children aged 6–23 mo	Children aged 24–47 mo	Total	Mean MUAC ± SD	Mean length/height ± SD
1	11	20	28	48	13.6 ± 1.1	80.7 ± 9.3
2	11	17	31	48	14.6 ± 2.8	82.6 ± 11.0
3	12	28	20	48	13.6 ± 1.2	78.5 ± 11.1
4	11	19	29	48	13.8 ± 0.9	80.3 ± 10.0
5	12	26	22	48	13.6 ± 0.9	80.4 ± 11.6
6	10	13	35	48	13.9 ± 1.1	85.6 ± 9.6
7	12	21	27	48	13.5 ± 1.2	82.3 ± 10.7
Total/all	79	144	192	336	13.6 ± 1.8	81.5 ± 10.6

1 MUAC, midupper arm circumference.

### Intraobserver precision

**FIGURE 1:**
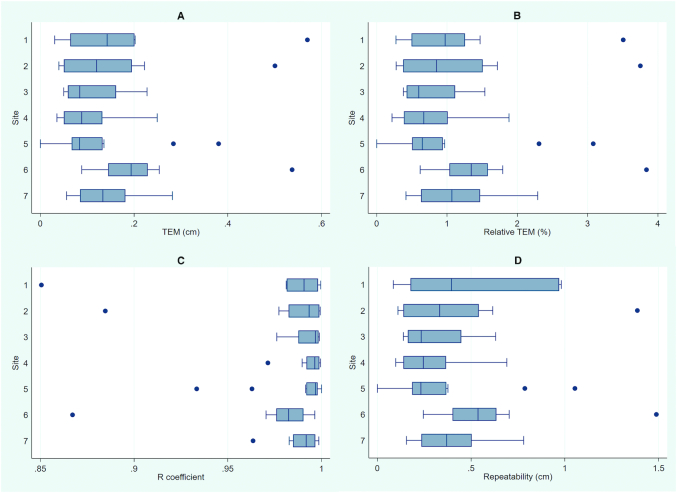
Box plots of intraobserver precision of MUAC measurements. Plots summarize precision by standardization site (*n* = 7) and by metric: TEM (A), relative TEM % (B), *R* coefficient (C), and repeatability (D). MUAC, midupper arm circumference; TEM, technical error of measurement.

Intraobserver TEM for measures of MUAC calculated among all observers (*n* = 79) ranged from a low of 0.00 cm to a high of 0.57 cm. *R* coefficients ranged from 0.851 to 1.000; therefore, the highest proportion of variation caused by measurement error was 15% and the lowest was <1%. Of trainees, 95% achieved *R* >0.95. Repeatability ranged from 0.00 to 1.49 cm. Of the higher, 2 measurements taken by the observer on the same child had differences of ≤1.49 cm for 95% of subjects. Box plots for intraobserver precision present summary data by standardization site (**Figure 1**).

**FIGURE 2:**
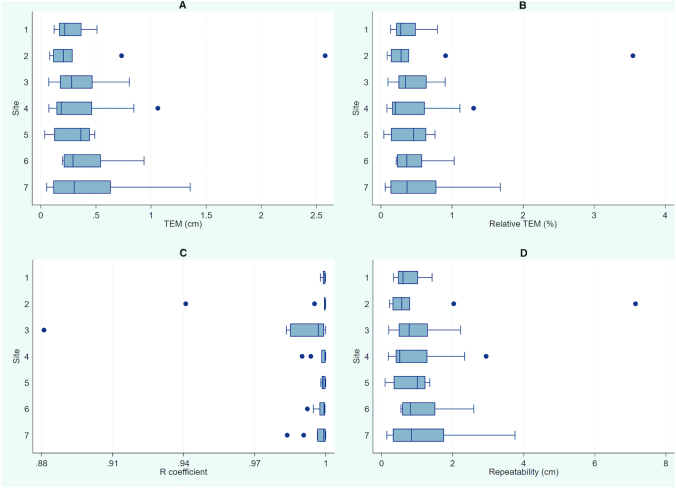
Box plots of intraobserver precision of length/height measurements. Plots summarize precision by standardization site (*n* = 7) and by metric: TEM (A), relative TEM % (B), *R* coefficient (C), and repeatability (D). TEM, technical error of measurement.

Intraobserver TEM for length/height was between 0.04 and 2.58 cm. *R* coefficients ranged from 0.881 to 1.000; at highest, 12% of variance was caused by measurement error, and at lowest <1% was caused by measurement error. Of trainees, 97% achieved *R* >0.95 for length/height measures. Repeatability ranged from 0.10 to 7.14 cm. Box plots present summary data (**Figure 2**).

**FIGURE 3:**
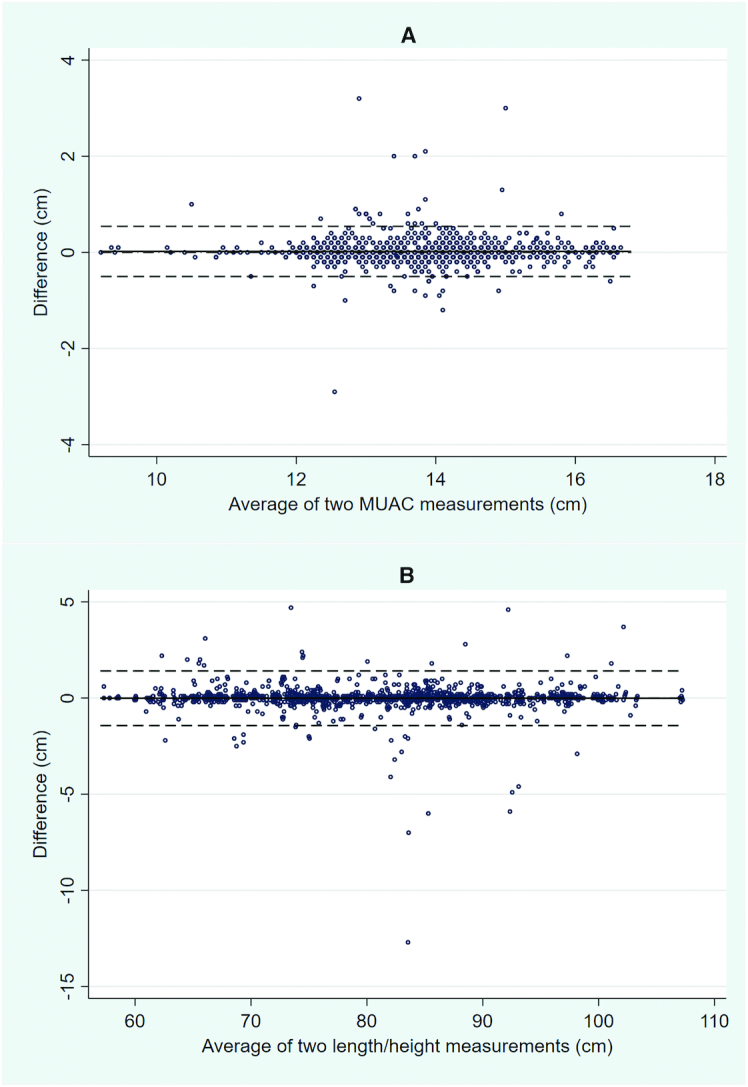
Bland–Altman plots for intraobserver measurements of MUAC (A) and length/height (B). Differences in measurement (*y* axis) are plotted against the average of the 2 measurements (*x* axis) taken by the same observer. Dashed lines represent the 95% limits of agreement (mean difference ± 2 SD). MUAC, midupper arm circumference.

Bland–Altman 95% limits of agreement for 2 different MUAC measurements on the same child taken by the same observer were −0.50 cm (95% CI: −0.53, −0.48 cm) to 0.54 cm (95% CI: 0.52, 0.57 cm) and for length/height were −1.43 cm (95% CI: −1.50, −1.36 cm) to 1.41 cm (95% CI: 1.34, 1.48 cm). Therefore, 5% of MUAC measurement pairs were different by more than ∼0.5 cm, and 5% of length/height measurement pairs were different by more than ∼1.4 cm (**Figure 3**).

### Interobserver precision

Interobserver precision was variable among the 21 teams. One group had the highest recorded precision metrics for both MUAC (TEM: 0.09; relative TEM: 0.64%; *R*: 0.99; repeatability: 0.24) and length/height (TEM: 0.06; relative TEM: 0.07%; *R*: 0.99; repeatability: 0.16). Another group also had the lowest overall precision for both MUAC (TEM: 0.45; relative TEM: 3.06%; *R*: 0.92; repeatability: 1.20) and length/height (TEM: 2.98; relative TEM: 3.49%; *R*: 0.92; repeatability: 8.25). Among teams, 90% had interobserver *R* >0.95 for MUAC and 95% for length/height. Interobserver precision of all anthropometry teams is presented in **Table 2**.

**TABLE 2:** Interobserver precision of MUAC and length/height measurements of 21 groups of observers during anthropometry standardization sessions at 7 sites^1^

Site and team		MUAC^2^				Length/height^2^			
Site no.	Team no. (*n* = observers, children)	TEM (cm)	Relative TEM (%)	*R*	Repeatability	TEM (cm)	Relative TEM (%)	*R*	Repeatability
1	1-1 (*n* = 4, 16)	0.22	1.57	0.977	0.62	0.40	0.48	0.999	1.11
1	1-2 (*n* = 3, 16)	0.15	1.17	0.989	0.42	0.33	0.41	0.999	1.00
1	1-3 (*n* = 4, 16)	0.42	3.09	0.919	1.16	0.26	0.34	0.999	0.72
2	2-1 (*n* = 4, 16)	0.18	1.29	0.985	0.50	1.04	1.04	0.990	2.87
2	2-2 (*n* = 3, 16)	0.29	2.03	0.960	0.82	0.14	0.17	1.000	0.40
2	2-3 (*n* = 4, 16)	0.10	0.70	0.995	0.28	0.49	0.59	0.998	1.35
3	3-1 (*n* = 4, 16)	0.19	1.40	0.983	0.54	0.38	0.46	0.999	1.04
3	3-2 (*n* = 4, 16)	0.11	0.80	0.995	0.29	0.37	0.47	0.999	1.02
3	3-3 (*n* = 4, 16)	0.28	2.11	0.963	0.79	0.56	0.74	0.997	1.56
4	4-1 (*n* = 4, 16)	0.14	1.03	0.991	0.39	0.64	0.64	0.996	1.78
4	4-2 (*n* = 4, 16)	0.18	1.30	0.984	0.51	0.11	0.14	1.000	0.31
4	4-3 (*n* = 3, 16)	0.09	0.64	0.997	0.24	0.06	0.07	1.000	0.16
5	5-1 (*n* = 4, 16)	0.11	0.79	0.995	0.30	0.27	0.34	0.999	0.76
5	5-2 (*n* = 4, 16)	0.16	1.22	0.988	0.46	0.37	0.46	0.999	1.03
5	5-3 (*n* = 4, 16)	0.16	1.18	0.988	0.45	0.64	0.81	0.996	1.78
6	6-1 (*n* = 4, 16)	0.43	3.06	0.913	1.20	2.98	3.49	0.921	8.25
6	6-2 (*n* = 4, 16)	0.27	1.89	0.967	0.74	0.75	0.86	0.995	2.07
6	6–3 (*n* = 2, 16)	0.21	1.56	0.980	0.58	0.75	0.88	0.995	2.07
7	7-1 (*n* = 4, 16)	0.10	0.74	0.995	0.27	0.37	0.43	0.999	1.02
7	7-2 (*n* = 4, 16)	0.21	1.54	0.980	0.58	1.19	1.51	0.987	3.31
7	7-3 (*n* = 4, 16)	0.23	1.70	0.976	0.64	0.23	0.28	1.000	0.65
	All, mean ± SD (*n* = 21, 336)	0.20 ± 0.10	1.47 ± 0.68	0.977 ± 0.02	0.56 ± 0.27	0.59 ± 0.62	0.70 ± 0.72	0.994 ± 0.02	1.63 ± 1.71

1 MUAC, midupper arm circumference; TEM, technical error of measurement.

2 Based on the Multicentre Growth Reference Study, WHO recommends maximum allowable differences between measurements of 0.5 cm for circumferences and 0.7 cm for length/height (4).

## Discussion

Precise data are necessary to assess the effectiveness of nutrition interventions at scale, particularly those targeting stunting or wasting. However, without careful training and quality control measures, measurement error may result in poor cross-survey comparisons and limited ability to detect intervention impacts.

We documented methods to conduct anthropometric standardization among trainee anthropometrists and their field supervisors undergoing training for a household nutrition survey. Each trainee twice measured a total of 16 children, and the same children were measured by all team members to provide estimates of intraobserver precision. Results were immediately calculated to examine precision and to enable retraining of staff found to have low precision relative to that of others.

We showed that the majority of trainee anthropometrists were able to achieve reasonable precision in MUAC and length/height measurements. Our results are consistent with those of studies using anthropometrists drawn from a pool of health professionals or other highly educated groups, in which *R* coefficients of ≥0.95 were achieved (2). However, our results are also similar to those of a study in Ethiopia in which community-drawn anthropometrists achieved interobserver TEMs of 0.22 MUAC and 0.67 length/height, which are comparable to interobserver TEMs presented in Table 2 (10).

Standards for acceptable precision have not been agreed, but Frisancho (21) proposed reference levels of 0.70 intraobserver and 0.95 interobserver TEM for height. We found that 95% of intraobserver and 86% of interobserver length/height TEMs were below those values. Reference levels of 0.35 cm intraobserver and 0.43 cm interobserver TEM were proposed for MUAC (21). In this study, 95% of interobserver and 95% of intraobserver TEMs had lower values.

A few anthropometrists recorded measurements with much larger variability than the average. This was depicted in the Bland–Altman plots, which showed that 95% of intraobserver differences between measurements were less than ∼1.4 cm for length/height—a value that is nevertheless more than twice that of the maximum acceptable difference of 0.7 cm between 2 separate length or height measures as specified by the SURE baseline survey protocol (22). (If differences exceeded this value, a third measurement was required to be taken.) Such large differences may be the result of poor measurement or reading technique or may reflect errors in the recording process. Either way, the data taken and analyzed during the standardization exercise were useful to retrain selected anthropometrists and to ensure maximal skill in both measurement and recording.

### Limitations

Precision of measurement is only 1 aspect of quality control for anthropometric measurement, and due to resource and personnel limitations, accuracy could not be assessed as part of the standardization exercise. Experienced or criterion anthropometrists were unavailable in sufficient numbers (1). Therefore, intraobserver variability was analyzed within teams of 2–4 trainees rather than between trainees and experts.

Similarly, we were unable to assess reliability of measurements. It has been recommended that standardization exercises take measurements at 2 different time points (23). In the Ethiopian context, however, the difficulties of recruiting, managing, and retaining such a large number of children are nontrivial. Caregivers committed hours both in travel to health posts and participation. It was deemed infeasible to attempt to retain children for a second measurement at a separate time point, and therefore all children were doubly measured within 1 morning or 1 afternoon. Despite efforts to mitigate trainee recall, this increased the risk of lower measurement independence.

Due to convenience sampling, the overall age distribution of children assigned to anthropometry groups was variable (Table 1). Higher measurement variability has been observed in younger and smaller children compared with older and larger children (10) and also between measurements taken lying down (length) and standing up (height) (24). Data on type of length measurement were not recorded, which precluded differentiation between length and height measures in these analyses, despite the potential for different errors. In future standardization processes, we recommend that the type of length measurement be recorded for separate analysis.

### Conclusions

Large numbers of anthropometrists (>75) in low-income settings can attain satisfactory precision in anthropometry following training and standardization. Such protocols permit researchers to assess trainees, identify individuals who have not achieved the desired level of precision, and retrain or adjust roles prior to survey deployment.

## Supplementary Material

nzaa139_Supplemental_File

## Notes

This study was supported by the Children's Investment Fund Foundation. The funders did not participate in the study design, data collection or analysis, review of the manuscript, or decision to publish.

Author disclosures: The authors report no conflicts of interest.

Supplemental Table 1 is available from the “Supplementary data” link in the online posting of the article and from the same link in the online table of contents at https://academic.oup.com/cdn/.

Abbreviations used: EPHI, Ethiopian Public Health Institute; MUAC, midupper arm circumference; SURE, Sustainable Undernutrition Reduction in Ethiopia; TEM, technical error of measurement.
